# Practical issues in picture archiving and communication system and networking

**DOI:** 10.4103/0971-3026.59743

**Published:** 2010-02

**Authors:** Arjun Kalyanpur, Jasbir Singh, Ricky Bedi

**Affiliations:** Chief Radiologist, Teleradiology Solutions, Bangalore, India

**Keywords:** Computers, networks, picture archiving and communication system, teleradiology

## Abstract

Picture Archiving and Communication System (PACS) is a key workflow tool in the functioning of radiology departments worldwide, today, and its utilization is rapidly growing in India. The key challenges in PACS implementation are related to vendor and feature selection, integration with the existing HIS, user training, maintenance and scalability to meet increasing demands. Additionally, the networking requirements that PACS imposes on hospital networks are not insignificant. This article attempts to review these issues from the standpoint of what a prospective or new user needs to know.

## Introduction

PACS (picture archiving and communication systems) have been in existence for several years and have become an integral part of the infrastructure of radiology and imaging departments across the world.[1–5] However, although the core technology continues to evolve and improve, the key practical issues involved in the implementation of a PACS over an internal LAN (local area network)-with the associated maintenance, troubleshooting, training, and integration issues-still remain and, if anything, continue to increase in complexity as our dependence on PACS grows.

To make matters more complex, the closely associated field of teleradiology involves seamlessly integrating a plethora of varied PACS across locations, indeed across continents. This scenario also involves addressing and overcoming issues related to widely varied networks, creating secure VPN or virtual private network tunnels, configuring multiple firewalls, as well as establishing and testing DICOM (digital imaging and communications in medicine) transfers. This article attempts to review the key practical issues and challenges involved in PACS [Table 1] and networking, as they manifest both in the hospital as well as in the teleradiology environment.

**Table 1:** List of core issues in practical implementation of a picture archiving and communication system

Procurement and installation
Networking and integration
Training
Troubleshooting
Maintenance
Upgrading/switching vendors

## Networking Issues

**Figure 1:**
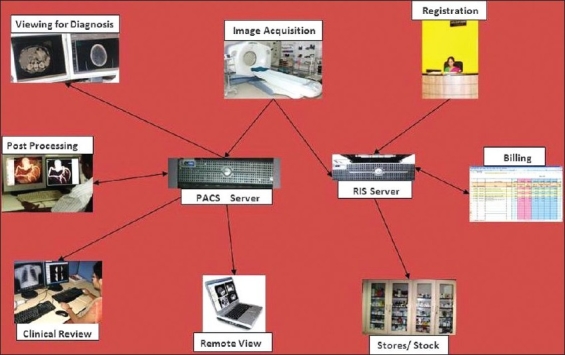
The Picture archiving and communication system enterprise extends through the entire hospital system and spans all radiology related functions

As illustrated in Figure 1, networking of a complete PACS system across a healthcare enterprise allows for the maximum impact of PACS to be felt across several departments. Given that cost is a critical factor in PACS deployment, it is important to consider the relatively lower incremental cost of an enterprise PACS solution that extends through the entire hospital system as opposed to having standalone subunits in each department or facility.

The major components in the PACS workflow domain include acquisition; transfer to a central repository, with appropriate archiving; remote access for viewing; reporting; support in clinical decision-making (as with surgical planning); billing; and accounting; etc. All these components have to be supported by a robust and secure network, which is the backbone of the PACS.[6] In today's scenario, networking should typically be using CAT 6 cabling. Organizations that implement PACS on an existing CAT 5 network, risk significant slowdown in data transmission down the line with burgeoning PACS data. The utilization and appropriate configuration of appropriate routers, switches, and firewalls is equally critical to the implementation of a secure and efficient PACS.

## Implementation of New Picture Archiving and Communication System

The challenges associated with the implementation of a new PACS begin with the selection process itself. Any PACS has to address the goals of higher productivity in the healthcare environment, quicker and more efficient decision-making, improved patient care, and also provide a reasonable return on investment by lowering costs. These goals have to be in synchronization with parameters of the PACS such as features, scale and scalability, cost and, quite significantly, post-implementation support. The same parameters apply for the hardware components. It is important to define the goals prior to procurement and to prioritize them in order to be clear on which optional items are imperative and which are dispensable.[7,8] This, to some extent, is dictated by the modalities. For example, a high-end 3D post-processing component is unlikely to be of significant value in a department which has a low-end CT scanner or which already owns a 3D workstation.

Once a PACS has been selected, procured, and installed, the next key milestone is integrating the same with various modalities for acquisition, with hospital information systems (HIS) and/or radiology information systems (RIS), and with peripherals for data backup and printing. This stage typically has problems associated with testing and fine-tuning of the DICOM transfers between the PACS workflow modules, compression adjustment for optimum speed of transmission, setting up of IT authentication and access permissions, and network hardening, etc.

It is at this stage that the compatibility issues typically surface, necessitating the development and implementation of an HL7 translator, which integrates the modalities and software involved. In the teleradiology environment, such a translator is especially important, given that the dialogue takes place between multiple disparate commercial PACS and RIS systems. Such a translator also helps to avoid manual re-entry of patient data from the PACS to the RIS, thus serving as an efficiency and productivity enhancing tool and reducing the turnaround times for radiology reporting. It needs hardly be emphasized that an experienced IT team is critical in addressing and solving these kinds of implementation and integration issues and hurdles in tandem with radiologist input. As end users, it is up to the radiologists using the technology to ensure optimal usage.

## Training of Users

**Figure 2:**
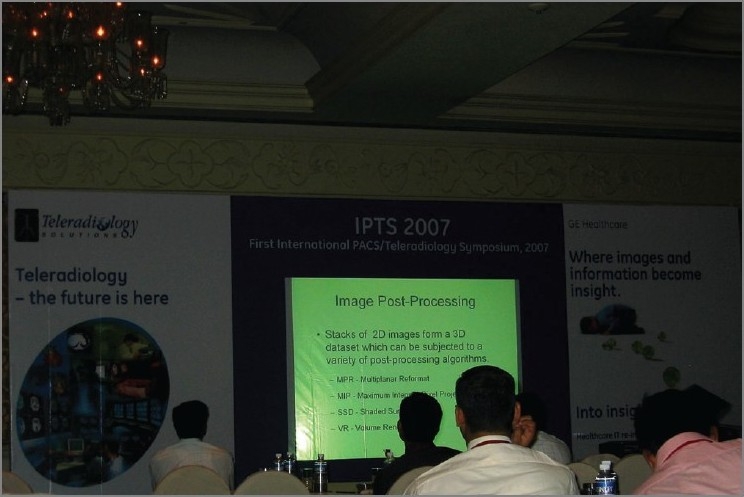
Training is an essential part of picture archiving and communication system implementation

PACS/RIS training is a very important practical aspect that can ultimately have a dramatic impact on the efficiency of the PACS in the long run and on the time taken for the return on investment to begin. It is imperative that the training of users begins even before the PACS are installed, so that PACS productivity starts from day one [Figure 2].

In addition to on-site face-to-face training, collaborative internet-based tools such as GoToMeeting and Netmeeting facilitate distance training by the developers and support staff in optimizing usage of technology by the radiologist. Periodic review sessions for radiologists are also of value in communicating updates and upgrading information.

## Troubleshooting and Maintenance Issues

Successful implementation of PACS only means that one stage in the PACS lifecycle has been completed, somewhat akin to the successful delivery of a newborn. After this we enter the equally critical phase of maintenance and increasing usage (the ‘toddler’ period) during which it is imperative from a workflow perspective that the setup continues to work with minimal disruption and downtime. This again involves having a dedicated, well-qualified, and well-trained PACS administrator and IT support team to address all eventualities. Periodic interaction between the in-house team and the vendor is crucial to optimal PACS utilization.

**Figure 3:**
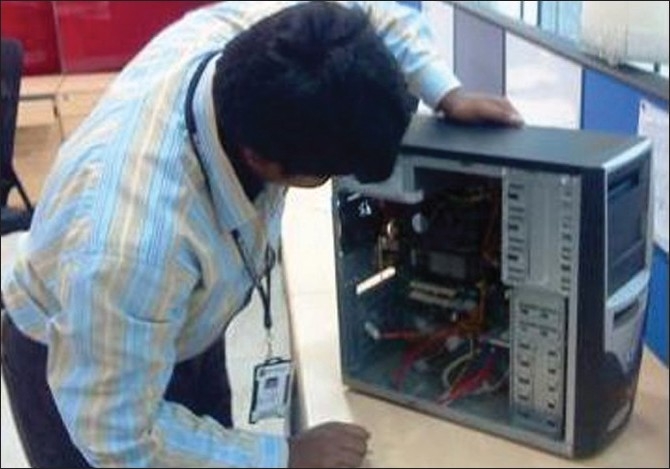
Hardware maintenance and periodic up gradation are keys to maintaining robust picture archiving and communication system infrastructure

Equally important is periodically scheduled preventive maintenance for both the application software as well as hardware. This includes, but is not limited to, points such as regularly clearing the viewer cache on workstations, defragmentation of workstation memory, updating antivirus definitions, and cleaning out the Windows registry [Figure 3]. PACS server preventive maintenance through annual maintenance contracts with the vendor is also important to ensure hardware uptime, given that hardware outages can be devastating to a network's infrastructure.

## Upgrading or Switching Vendors

There typically comes a stage in a radiology department's evolution when it becomes necessary to upgrade or revamp the IT infrastructure or move to a better PACS. This has the potential to be extremely traumatic, but it can be controlled if factored in at the initial planning stage itself. Key parameters from an IT perspective include: a) physical space for growth, b) redundant wiring for both power and networking, and c) archiving data for legacy migration (and of course as backup). From a PACS angle, this would involve addressing scalability[9] or, as is often the practical solution, taking a modular approach.

Switching to an altogether different PACS vendor due to reasons of obsolescence, inadequate performance or, occasionally, due to acquisition of the parent vendor company (with the resulting change in vendor-client relationship), is a more complex scenario. Typically, this involves re-training personnel, overcoming inherent resistance to a new application with it's own different GUI and commands, compatibility between the old and new PACS (important when comparing with prior studies), and migration of data. In such a scenario, the experience gained with the old PACS system is often valuable for setting expectations for the future. Thus, even this substantial cloud can be found to have a silver lining. A word of caution, however, is that some vendors may promise the sky in order to win over clients; so due diligence is particularly important at this stage, including site visits to existing client departments to hear the actual experiences on the ground, as opposed to merely viewing sales presentations.

## Conclusion

PACS and networking, both being rapidly evolving domains, have their sets of practical issues and challenges pre- and post-implementation. The key parameters that determine their optimal utilization are systematic planning, a well-qualified and experienced PACS administrator/IT department, periodic radiologist training, regular maintenance, and a readiness to upgrade and, if necessary, to switch to a more appropriate technology at the opportune time.
